# Improvement of an Edge-IoT Architecture Driven by Artificial Intelligence for Smart-Health Chronic Disease Management

**DOI:** 10.3390/s24247965

**Published:** 2024-12-13

**Authors:** William Alberto Cruz Castañeda, Pedro Bertemes Filho

**Affiliations:** Electrical Engineering Department, Santa Catarina State University, Joinville 89219-710, Brazil; pedro.bertemes@udesc.br

**Keywords:** smart health, edge computing, chronic disease management, artificial intelligence, smart city application

## Abstract

One of the health challenges in the 21st century is to rethink approaches to non-communicable disease prevention. A solution is a smart city that implements technology to make health smarter, enables healthcare access, and contributes to all residents’ overall well-being. Thus, this paper proposes an architecture to deliver smart health. The architecture is anchored in the Internet of Things and edge computing, and it is driven by artificial intelligence to establish three foundational layers in smart care. Experimental results in a case study on glucose prediction noninvasively show that the architecture senses and acquires data that capture relevant characteristics. The study also establishes a baseline of twelve regression algorithms to assess the non-invasive glucose prediction performance regarding the mean squared error, root mean squared error, and r-squared score, and the catboost regressor outperforms the other models with 218.91 and 782.30 in MSE, 14.80 and 27.97 in RMSE, and 0.81 and 0.31 in $R^{2}$, respectively, on training and test sets. Future research works involve extending the performance of the algorithms with new datasets, creating and optimizing embedded AI models, deploying edge-IoT with embedded AI for wearable devices, implementing an autonomous AI cloud engine, and implementing federated learning to deliver scalable smart health in a smart city context.

## 1. Introduction

The increase in the urban population and the prevalence of non-communicable diseases (NCDs) are some of the problems facing any society worldwide. Fostering healthy habits among the population, proper treatment, and prevention measures are challenges for the health sector in any city. A World Bank Group report [1] shows that, today, 56% of the world’s population lives in cities, and this trend is expected to continue with the urban population growing to 70% by 2050. In the case of NCDs, cardiovascular diseases, cancer, chronic respiratory diseases, and diabetes are among the world’s causes of premature deaths. In 2008, more than 36 million people died from NCDs, including 14 million between the ages of 30 and 70. According to World Health Organization (WHO) projections, the total annual number of deaths from NCDs will increase to 55 million by 2030 [2]. Looking at these statistics reveals that the development of a viable infrastructure and services to address population health needs is required in cities.

NCDs are long-term health conditions whose management is particularly important in terms of costs and a person’s quality of life. They demand the transformation of healthcare from a traditional hospital-centered health management model to a new patient-centered model that pays more attention to self-management and emphasizes real-time self-monitoring with feedback on health data and patients’ well-being [3,4,5].

On the other hand, the smart city concept has gained remarkable traction globally, driven by the increasing interest in employing digital technologies to address urban challenges. In the last two decades, this concept has been developing research for several innovative services that enhance and stimulate sustainable development and growth [6,7]. A smart city is defined as a city that integrates technology and the natural environment to enhance the effectiveness of processes in every field of the city functioning to achieve sustainable development, safety, and health of citizens in order to increase the life standard [8]. Particularly in the healthcare domain, a smart city proves to be transformative, utilizing an extensive array of technological tools and processes to improve healthcare accessibility, optimize patient outcomes, reduce costs, and enhance overall efficiency [9]. Therefore, a predominant healthcare perspective on a smart city relates to the role of technological infrastructure in providing interconnected solutions.

To confront the increasing number of chronic patients and their long-term support, a smart city embraces health development with a technological approach focused on patient-centered care and personalized health planning to improve healthcare services and make them affordable for everyone [10]. The natural domain application of a smart city health development is known as smart health (s-health), which enables the personalization and introduction of intelligent management systems that can support the digital collection, processing, storage, transmission, and analysis of patient data, for example, to cater to remote diagnosis and the treatment of patients, or allow for the effective monitoring of public health [11,12].

According to [12], s-health defines the provision of health services using a context-aware network and sensing infrastructure for smart cities. Thus, the dynamic environment provided via s-health involves emerging technologies such as the Internet of Things (IoT), cloud computing-related technologies, and artificial intelligence (AI) to provide data-driven personalized care [13,14,15,16,17,18,19,20,21]. The studies of [22,23] show that the healthcare cost of chronic disease management decreases when s-health-related technologies are implemented. Research works such as [24,25] have developed s-health services to monitor and prevent NCDs and deliver long-term and integrated care management for a specific disease with AI techniques for inspection and retrieving data patterns. Nonetheless, according to [26], it was estimated until 2021 that a smart city application would generate 850 ZB of data from all people, machines, and things. Approximately 10% (7.2 ZB) of that data would be useful, stored, or used. Thus, to provide conditions for scalable s-health solutions, edge computing takes computational power as close as possible to the data source at the edge of the network, focusing on the IoT side.

In this context, this motivates us to propose a widespread solution for a smart city environment with an edge-IoT architecture driven by AI as an s-health application for chronic disease management. The proposed architecture consists of a wearable edge-IoT device, edge node, and cloud computing services to enable data-driven AI personalized care. A case study is presented in which edge computing further illustrates the vision of the proposed architecture to stretch the s-health activities to directly impact residents and become more socially relevant. In this way, the main contributions of the paper are as follows:

- Propose a decentralized approach that allows integrated care and long-term management for chronic non-communicable diseases to improve in real-time capabilities;
- Establish an architecture based on the principles of IoT, edge computing, and AI to develop an ecosystem that allows pre-processing at the edge of the network and data analysis in the cloud;
- Build a wearable edge-IoT device that captures and non-invasively pre-processes the parameters of oxygen concentration, pulse rate, skin temperature, and bio-impedance;
- Propose and establish a baseline of AI regression models to compare the non-invasive glucose predictive findings with a capillary blood glucose measurement.

The rest of the paper is organized as follows. Section 2, presents recent related works reviewing the identification of s-health services and their technologies. Section 3 describes the proposed architecture driven by AI for chronic disease management. Section 4 describes the study case of preliminary non-invasive diabetes prediction and the results. Section 5 presents the discussions, conclusions, and future directions.

## 2. Related Works

A smart city is considered an innovative system that will communicate with people and smart infrastructure embedded in a city. Defining key technologies to support s-health, ref. [27] describes health services as intelligent infrastructure using wearable devices, IoT, mobile internet, cloud computing, 5G communication, microelectronics, and AI for actively managing and responding to medical ecosystem needs. Thus, ref. [28] reviews the current trends of s-health systems and applications for smart cities. Those systems include smart home care for elderly or disabled people, smart wearable devices, smart textiles, and smart implantable devices. Ref. [29] propose an IoT-based automated patient s-health monitoring system for smart cities that consists of five components: data collection, report generation, hospital care, pharmacist care, and diagnostics. Ref. [30] analyzes different types of software architecture to develop an IoT system in the fields of smart cities, healthcare, and agriculture. The systematic review of [31] identifies the challenges, opportunities, and applications in edge AI for connected s-health in smart cities. On the other hand, the review of [32] presents how AI-powered IoT and wireless sensor networks are applied in s-health applications to understand their role in smart cities. Ref. [33] present a comprehensive survey of IoT-based devices, cloud, and edge-based s-health and AI. The survey of [34] analyzes existing edge computing architectures and techniques for s-health. A general edge/fog computing-based approach implements an architecture with three basic levels: edge, fog nodes, and cloud processing.

The work of [35] highlights the benefits of edge technology with AI and classifies s-health solutions via three viewpoints: IoT application, AI deployment edge location, and adopted AI technique. Moreover, a proposed s-health solution has three main layers: the sensor layer, the edge layer, and the cloud layer. The research of [36] investigates the benefits of using IoT, machine learning (ML), deep learning (DL), and edge computing to enhance s-health applications. The authors describe the general architecture of an IoT-based s-health system with three layers (perceptual, network, and cloud). Ref. [37] discusses the current state of the art of s-health, highlighting wearable and smartphone devices for health monitoring and ML for disease diagnosis. They also present an integrated software architecture for s-health with data analytics and AI tools. A general architecture for IoT focused on s-health monitoring presented by [38] includes three different levels. At the edge level, portable devices perform preprocessing and data acquisition. In the fog level, servers/gateways gather data from edge devices and perform local processing and storage. At the cloud level, computing tasks call services. Ref. [39] discusses an architecture for IoT and ML consisting of three levels: the edge level for preprocessing by devices; the fog level for data acquisition from sensor networks and edge devices; and the cloud level involves services for high-level computing tasks.

## 3. Materials and Methods

**Figure 1:**
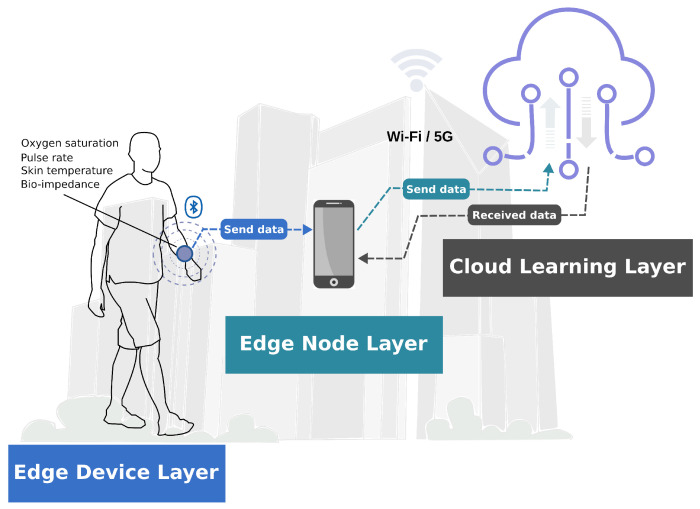
Proposed edge-IoT architecture organized into three layers: edge device, edge node, and cloud learning.

This section explains the fundamental layers of designing the edge-IoT architecture. As shown in Figure 1, the high-level view of the proposed architecture consists of three fundamental layers called the edge device layer, edge node layer, and cloud learning layer to enable data-driven AI personalized care.

### 3.1. Edge Device Layer

The edge device layer (EDL) was designed with a homemade bio-impedance wearable multi-parametric meter prototype device that sens and acquires non-invasively heterogeneous physiological data for preliminary local preprocessing and transmission. The edge-IoT device in this layer is a pivotal component of s-health applications. This device integrates a low-energy Bluetooth communication module for data transmission and sensors for the proactive measurement of oxygen concentration (Spo2), pulse rate (pr-bpm), and skin temperature, as well as 32 modules and phases of bio-impedance (BIA) into a frequency range from 0.1 to 100 kHz.

### 3.2. Edge Node Layer

The edge node layer (ENL) plays a role as a broker between EDL and the network. This layer has computing and caching capabilities to provide high-quality Wi-Fi or 5G network connections and computing services near EDL. Thus, the edge node receives data from the EDL that are initially converted into a standard JSON format.

### 3.3. Cloud Learning Layer

The final layer, the cloud learning layer (CLL), contains an inner AI–cloud engine, which includes three components interconnected in a pipeline: data handling (DH), model, and inference. The DH component receives the data from the ENL in JSON format and stores them internally in a database in the cloud. DH also involves scaling and selection techniques over selected and extracted relevant features. The model component applies the selected relevant features and the training of an appropriate AI model. The inference component is responsible for making predictions on new unknown input data. Inference does not reevaluate the model, apply knowledge from the training, or use it to infer a result. Finally, the inference outcomes are stored in the database and transmitted to the ENL. Thus, the AI–cloud engine pulls the data, processes it, trains a model, and presents a result that interacts with the ENL for further requests.

#### 3.3.1. AI Algorithms Baseline

There are several works of published literature on glucose prediction that use machine learning and deep learning algorithms in the context of s-health. Studies implement auto-regressive (AR), linear regression (LR), support vector regression (SVR) with Gaussian kernel, naive Bayes (NB), multi-layer perceptron neural network regression (MLP), Gaussian process regression (GPR), random forest regression (RFR), k-nearest neighbor regression (KNN-R), and one-dimensional convolutional neural networks (CNN-1D) [40,41,42]. Therefore, this research establishes a baseline of twelve supervised AI regression models for glucose prediction. Within the scope of this research, the baseline algorithms are multi-linear regression (MLR), SVR, KNN-R, decision tree regressor (DTR), bagging DTR (B-DTR), adaboost regressor (AB-R), xgbBoost regressor (XGBR), RFR, gradient boosting regressor (GB-R), catboost regressor (CB-R), MLP, and CNN-1D.

Thus, the model component contains the AI algorithm with the best performance evaluation of this baseline and applies training to it. Performance evaluation is accomplished offline, in an AI algorithm baseline outside the AI–cloud engine. In the inference component, the trained AI algorithm makes predictions on new unknown input data, and the results are stored in the database and transmitted to the ENL when requested.

#### 3.3.2. AI Algorithm Evaluation

For each regression AI algorithm in the baseline, well-known regression metrics were used, including the mean square error (MSE), root mean squared error (RMSE), and coefficient of determination ($R^{2}$). The MSE ranks the performance of the model in the prediction problem. The values fall in the range [0, ∞], and smaller values indicate better model performance. A smaller RMSE value implies a model for better predictions. $R^{2}$ coefficient values fall in the range [0, 1], and larger values indicate better model performance.

## 4. Results

The WHO 2014 statistics emphasize that there are about 422 million diabetes patients worldwide, the majority living in low–middle-income countries, and in 2019, 1.5 million deaths were directly attributed to diabetes each year [43]. Additionally, the International Diabetes Federation (IDF) estimates that, in 2021 [44], 537 million people had diabetes, and over 6.7 million people aged 20–79 would have died from diabetes-related causes. The projections reach 643 million by 2030 and 783 million by 2045. Two other causes for alarm raised by the IDF are the increased number of children and adolescents living with diabetes (over 1.2 million have type 1 diabetes) and the high percentage (45%) of people with undiagnosed diabetes type 2. Thus, using the proposed edge-IoT architecture, a case study presents an initial work to put forward an s-health application for measuring glucose noninvasively.

### 4.1. Data Source

**Figure 2:**
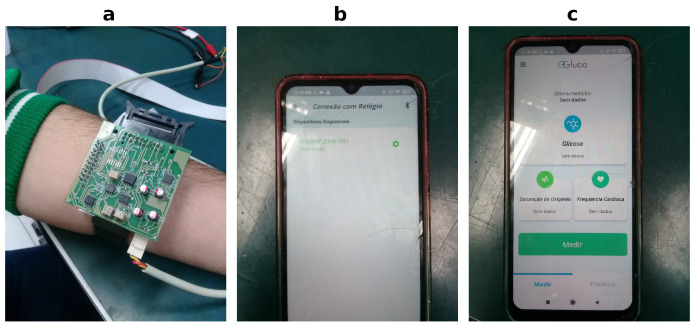
Wearable edge-IoT multi-parametric prototype or EDL (**a**) for data acquisition of oxygen concentration, pulse rate, skin temperature, and bio-impedance. (**b**) ENL paired with the homemade wearable prototype. (**c**) ENL stores the data temporarily, transforms it, and sends it to the CLL.

The data used in this research derive from the EDL illustrated in Figure 2a. A multivariate dataset was created with 20 healthy volunteers. Measurements were taken every 15 min for 2 h with the volunteers. At the same time, capillary blood glucose (CBG) was collected with a digital glucometer (^®^Accu-Check Guide, Roche, Indianapolis, IN, USA). All the volunteers fasted for 12 h and then consumed a liquid substance containing 75 g of glucose to assess the glycemic response. The multivariate dataset with 172 instances was assembled with 68 attributes available on it that capture characteristics of CBG, oxygen concentration (Spo2), pulse rate (pr-bpm), skin temperature (temp), and into a frequency range from 0.1 to 100 kHz 32 modules and 32 phases related to bio-impedance (BIA). Figure 2a shows the EDL layer of the proposed architecture performing non-invasive sensing and data acquisition. The custom design of this device allows for carefully choosing every component to achieve the best performance. Another advantage is the full control of every aspect related to custom single-frequency and multifrequency waveform excitation signals, digital filters, and other features that can be performed with a microcontroller. The dataset with 172 instances was handled with data cleaning pre-proceeding tasks as missing values and removing outliers. In essence, these tasks remove data associated with eight volunteers. Thus, the dataset, after pre-processing, resulted in 104 instances from twelve volunteers.

**Figure 3:**
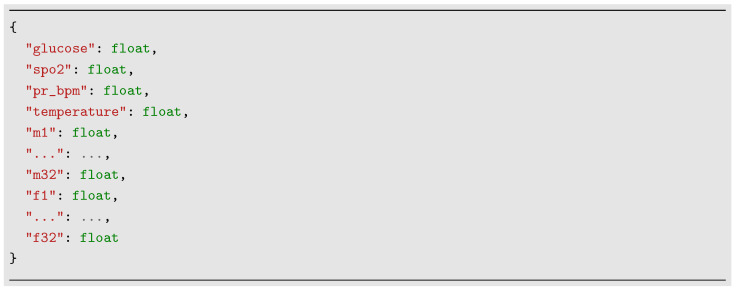
Standard JSON format with the acquired and stored data in the edge device.

When the ENL receives data from the EDL, those are in raw format. Figure 2b shows the ENL paired with the EDL, and Figure 2c ENL is ready for storing the data temporarily, as well as sending it to the next layer. Thus, with the data stored in the ENL, those are formatted as a standard JSON, as illustrated in Figure 3, where *glucose* corresponds to the CBG value, *spo2* corresponds to the oxygen concentration value, *pr_bpm* to the pulse rate value, *temperature* to skin temperature, and *m1* to *m32* modules and *f1* to *f32* to phases related to bio-impedance values.

Additionally, the ENL implements the device authorization through the OAuth 2.0 Authorization Framework before sending data to the next layer. The three main steps involved are requesting authorization from a user to access their data, querying the user’s data using the provided access token, and refreshing expired access tokens with the corresponding refresh token. Therefore, ENL renders results from the CLL because it can reply to EDL more quickly than the CLL. By deploying the edge node to perform these computation tasks, the processing can be accelerated while ensuring real-time capabilities. In addition, edge nodes have a cache, which can improve the response time by caching data from EDL. This design of edge layers (EDL and ENL) allows a network edge with 5G technology that offers wireless connectivity, achieving low latency and high cellular speed, which is particularly useful in cases where high responsiveness is required.

### 4.2. Feature Scaling and Selection

**Figure 4:**
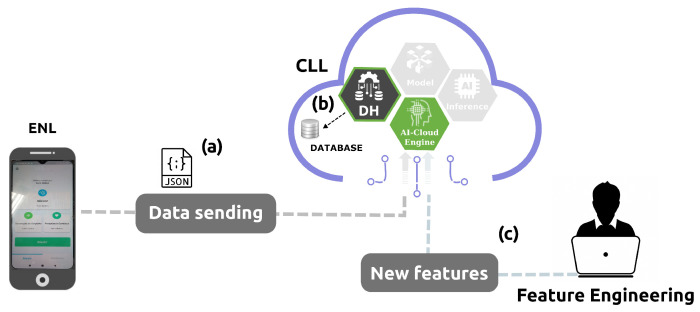
Edge node (ENL) sends JSON data to the CLL (**a**). DH component stores those data (**b**) and also receives new features. Feature engineering tasks such as scaling and selection are accomplished outside the CLL (**c**) and interact with the DH component.

As illustrated in Figure 4, with data temporarily stored and transformed in a JSON format, the ENL sends them to CLL (a), where the DH component stores it (b). Feature engineering tasks (scaling and selection) are accomplished offline outside the AI–cloud engine (c).

All the attributes in the dataset were handled for standardization. The scaling techniques used in this research to set numerical variables to similar value ranges are min-max normalization, standardization (z-score normalization), and robustness to outliers. The one that best fits the AI algorithm was chosen. After the data were scaled, feature selection techniques removed non-useful features to reduce the complexity of the model. This research implemented sequential feature selection (SFS) to avoid over-fitting and provide suitable performance for each AI algorithm. The SFS starts with ten features until completing sixty, incrementing in five units. In each round, a dataset with reduced features is obtained and split into training and testing sets with an 80–20% ratio. Grid, random, and Bayes search approaches accomplish hyperparameter optimization for AI algorithms at the baseline. The best-optimized hyperparameters are used with the reduced features to perform training and inference on each algorithm. For the deep learning CNN-1D algorithm, the topology and hyperparameters consist of a 1D convolution layer with 32 filters, a kernel size of 2, an input shape of 66, a ReLU activation function, a flatten layer, two dense layers with 64 and 1 units, respectively, and an Adam optimizer. With the new features obtained offline and set to the DH component, DH accesses the database, extracts those relevant features to apply the correct scaling technique, and transfers them to the model component to train an AI algorithm.

### 4.3. Performance of the Best AI Algorithms

**Figure 5:**
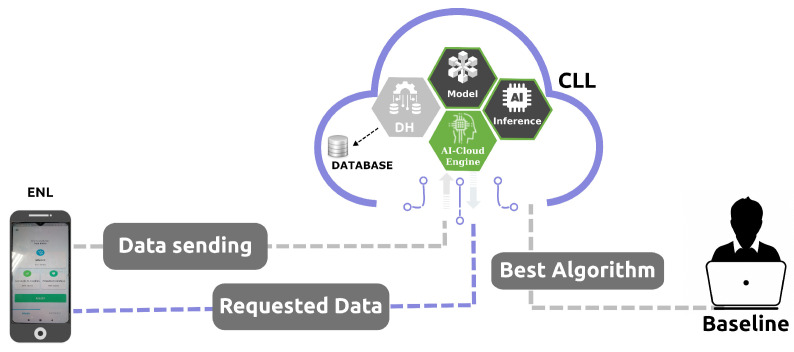
The model component includes the best and most appropriate algorithm for the inference component, which stores and implements predictions with new data. Thus, when appropriate, the ENL requests prediction data for user visualization.

With the 104-instance dataset, AI baseline algorithms were evaluated offline with the best features as inputs in both the training and test sets. The MSE, RMSE, and $R^{2}$ error metrics quantify the model performance in predicting glucose. Also, k-fold cross-validation, with k = 5, was used to evaluate each AI algorithm on an unseen data sample. Figure 5 shows the flow between the model and the inference components for training and inference tasks. It illustrates the interaction of the model component with the AI algorithm baseline and the interaction of the inference component with the ENL when data are requested.

**Figure 6:**
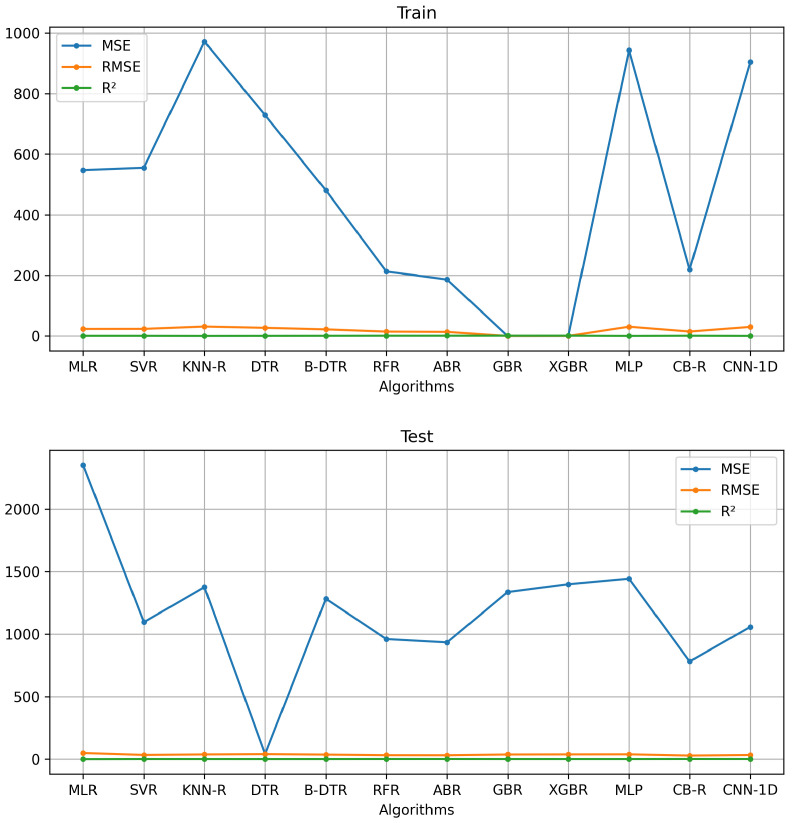
Baseline AI algorithms’ performance in training and test sets with reduced features.

Table 1 and Figure 6 show the performance outcomes of the best features with the AI algorithms’ baseline on the training and test sets. A comparison of the baseline AI algorithms elucidated that two ensemble methods (RFR and ABR), CB-R, and CNN-1D yielded slight improvement over the other algorithms. However, a comparison among these models revealed that CB-R outperformed with 218.19 and 782.30 in MSE, 14.80 and 27.97 in RMSE, and 0.81 and 0.31 in ($R^{2}$), respectively, on training and test sets. ABR is the second best with 186.19 and 934.03 in MSE, 13.65 and 30.56 in RMSE, and 0.84 and 0.18 in ($R^{2}$), respectively, on the training and test sets. RFR is the third best with 212.95 and 960.34 in MSE, 14.59 and 30.98 in RMSE, and 0.82 and 0.16 in ($R^{2}$), respectively, on the training and test sets.

**Figure 7:**
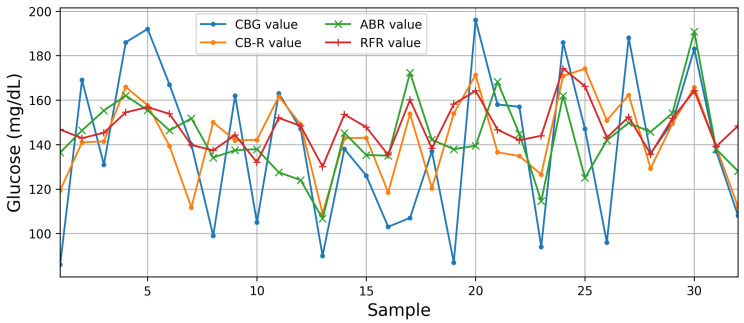
Predicted values of the CatBoost Regressor (CB-R), AdaBoost Regressor (ABR), and Random Forest Regressor (RFR) AI models compared to capillary blood glucose (CBG) values.

CB-R is a variant of gradient boosting on decision trees. It also uses an algorithm called the symmetric weighted quantile sketch (SWQS), which automatically handles the missing values in the dataset to reduce overfitting and improve the overall performance of the dataset. On the other hand, AdaBoost is a meta-estimator that begins by fitting a regressor on the original dataset and then fits additional copies of the regressor on the same dataset, but the weights of instances are adjusted according to the error of the current prediction; as a consequence, the subsequent regressors focus more on challenging cases. Random forest is a meta-estimator that fits several classifying decision trees on various sub-samples of the dataset and uses averaging to improve the predictive accuracy and control over-fitting. Therefore, ensemble methods combine the predictions of several base estimators built with a given learning algorithm to improve generalization and robustness over a single estimator. Figure 7 compares the CB-R, ABR, and RFR glucose prediction outcomes with CBG acquired for the digital glucometer.

Additionally, Table 2 shows the performance of the best AI algorithm of the baseline and compares it with similar approaches available in the literature. All of these approaches implement a regression task to predict, with non-invasive measurements, continuous glucose monitoring through wearable sensors. The evaluation metrics of the other algorithms are taken from the respective literature.

### 4.4. Performance of the Proposed Architecture

**Figure 8:**
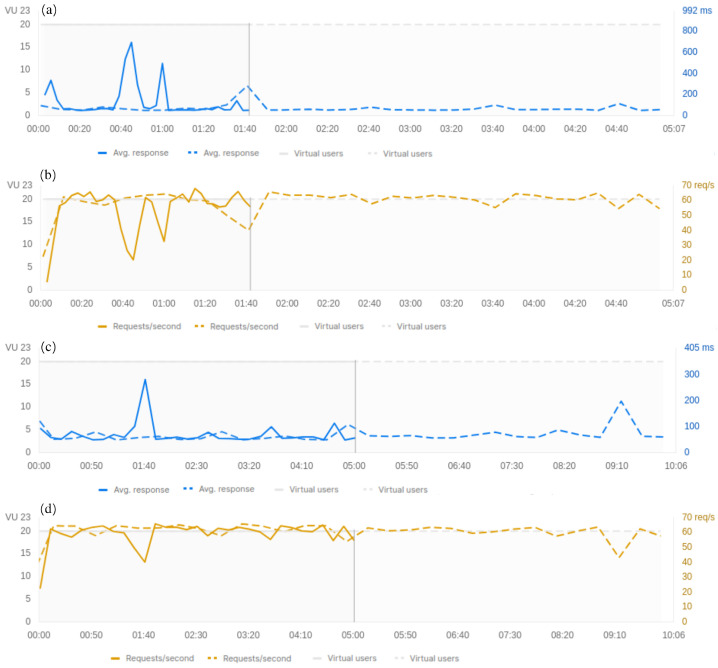
Performance test simulation with 20 virtual users acting concurrently with the CLL. Graphs (**a**,**b**) illustrate comparative results of the average response time and throughput between the 1:40 and 5-min time intervals. Graphs (**c**,**d**) illustrate comparative results for the average response time and throughput between the 5 and 10-min time intervals.

**Figure 9:**
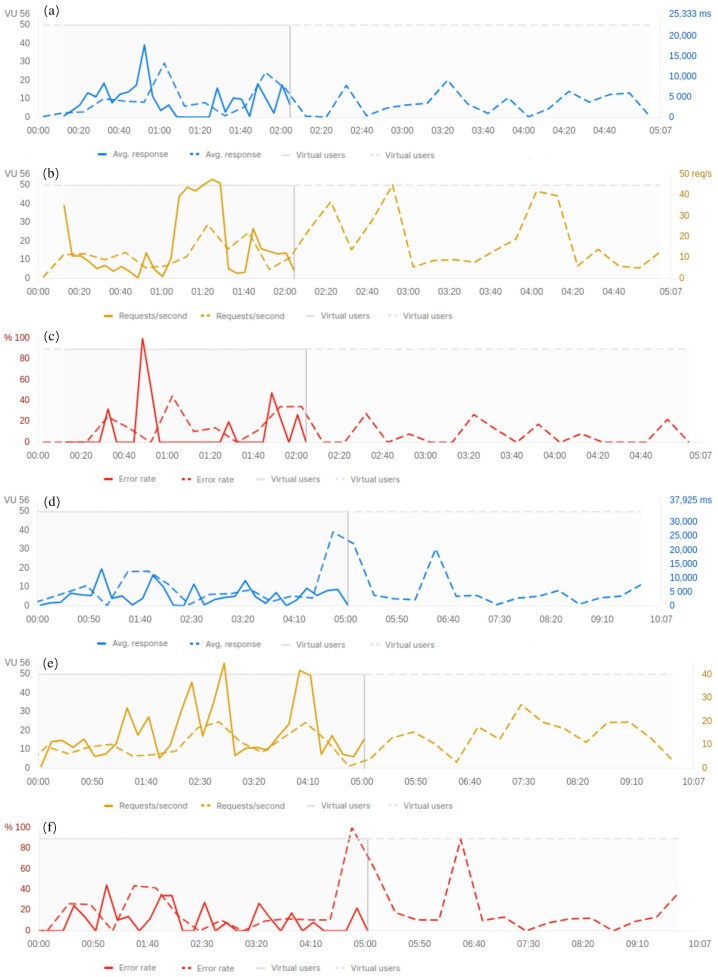
Performance test simulation with 50 virtual users acting concurrently between the ENL and the CLL. Graphs (**a**–**c**) illustrate comparative results for the average response time, throughput, and error between the 2 and 5-min time intervals. Graphs (**d**–**f**) illustrate comparative results for the average response time, throughput, and error between the 5 and 10-min time intervals.

Performance tests were carried out to simulate the activity of several users and evaluate the architecture in terms of the average response time, throughput, and error rate. Simulation experiments evaluated data transmission between virtual ENLs and the CLL through HTTPS protocol and over a 5G connection. Each virtual ENL user ran four requests into a repeating loop. Also, virtual ENLs worked in parallel to simulate the real-world load on the CLL. The simulation range was 1:40, 2, 5, and 10 min, and two configurations were implemented to put an increased load on the CLL as follows: (1) 1:40, 5, and 10 min with 20 fixed virtual ENLs, and (2) 2, 5, and 10 min with 50 fixed virtual ENLs. Figure 8 and Figure 9 show the architecture performance metrics under those two sets of simulations.

Figure 8 shows the architecture experiments’ results with 20 fixed virtual ENLs. Figure 8a,b depicts the average response time and throughput between 1:40 and 5 min, respectively. In the range of 1:40 min (blue and yellow lines, filled) were sent a total of 5401 requests; the average response time was 101 ms, and the throughput was 52.20 requests/s. In the range of 5 min (dotted blue and yellow lines) were sent a total of 18,126 requests; the average response time was 67 ms, and the throughput was 58.88 requests/s. Figure 8c,d depicts the average response time and throughput between 5 and 10 min, respectively. In the range of 10 min (dotted blue and yellow lines) were sent a total of 36,757 requests; the average response time was 67 ms, and the throughput was 60.63 requests/s. In this first simulation, the error rate was zero %. Thismeans that all requests were sent successfully and returned a 200 response code.

On the other hand, Figure 9 shows the architecture experiments results with 50 fixed virtual ENLs. Figure 9a–c depicts the average response time, the throughput, and the error distribution between 2 and 5 min, respectively. In the range of 2 min (blue, yellow, and red lines filled) were sent a total of 1819 requests; the average response time was 2520 ms, the throughput was 14.25 requests/s, and the error rate was 6.05%. In the range of 5 min (dotted blue, yellow, and red lines) were sent a total of 4738 requests; the average response time was 2669 ms, the throughput was 15.40 requests/s, and the error rate was 7.87%. Figure 9d–f depict the average response time, the throughput, and the error distribution between 5 and 10 min, respectively. In the range of 10 min (dotted blue, yellow, and red lines) were sent a total of 7223 requests; the average response time was 3737 ms, the throughput was 11.89 requests/s, and the error rate was 11.46%. This second simulation shows an error distribution over time for the requests and returns a 502 Bad Gateway response code.

## 5. Discussions and Conclusions

This work presents an edge-IoT architecture driven by AI to enable s-health solutions for a smart city. The proposed architecture connects a homemade edge-IoT wearable device to the cloud through an edge node for the real-time monitoring of physiological signals, and the AI–cloud engine enhances performance to deliver better predictions. The proposed study case acquires, selects, and trains physiological signal data to make an inference of glucose noninvasively. The purpose was to demonstrate the feasibility of making AI-based glucose prognosis available everywhere and for everyone without requiring invasive blood tests or visiting a hospital.

The vision is to provide accessible s-health services promoting the tangible concepts of a smart city. According to the obtained glucose prediction outcomes, ensemble, and deep learning algorithms could be promising, as observed in the preliminary results. CatBoost Regressor works well to predict glucose when there is not much training data, and it uses a combination of diverse data from different physiological sources (spo2, pr_bpm, temperature, and BIA). The selection and manipulation of the data features can boost the model performance by looking for a suitable feature to obtain new knowledge. Thus, the AI model’s performance is improved, and the results are more convenient.

It is important to address that one of the reasons why we proposed an edge-based architecture without fog layers is because we envision a practical future with a combination of AI and edge computing devices that allow the deployment of intelligent solutions on hardware devices and make decisions in real time. Another advantage over fog computing is that edge devices and AI do not need internet connectivity to analyze data in real time. However, with the 5G rollout and 6G communication coming soon, edge devices are becoming more present and eliminating the requirement for external fog servers. Edge devices could comprise numerous tasks and responsibilities, such as the collection, analysis, processing, transmission, routing, and storage of data passing between networks. Thus, 6G will be the platform for connected intelligence, where the mobile network will connect vast amounts of intelligent devices.

Regarding the proposed architecture, its originality lies in its multi-layered edge IoT and AI-driven approach that bridges edge computing, wearable devices, and cloud learning specifically for chronic disease management. The study case addresses the gap in scalable, real-time monitoring systems that provide predictive insights for chronic health conditions and offer an efficient alternative to traditional invasive methods. When comparing our proposed architecture to the previous studies, this research accomplished a practical case study on non-invasive glucose prediction monitoring, established and used twelve AI models as a baseline for glucose prediction, and provided experimental approaches to architecture and model performance testing.

- Ref. [29] proposes a system consisting of five components: patient data collection, the generation of patient primary reports, hospital patient care, pharmacist patient care, and diagnostics.
- Ref. [30] only proposes a software architecture for an IoT system for a healthcare application in a smart city context.
- Ref. [31], in the context of applications of edge-AI and healthcare in smart cities, is the only study to implement a quantitative study case proposing a cloud-based s-health monitoring model.
- Ref. [33] does not mention works for diabetes prediction and/or management.
- Ref. [32] is a literature review that discusses in detail how AI-powered IoT and wireless sensor networks are applied in the healthcare sector. The research is a baseline study to understand the role of the IoT in smart cities.
- Ref. [34] presents an overview of IoT-based healthcare. Three works concern diabetes. The first concerns type 2 diabetes detection and monitoring. The smart sensors used are heart rate, blood pressure, activity, and blood glucose sensors. The fog/edge device used is a laptop computer. The AI method adopted is a hybrid deep-learning model for type 2 diabetes prediction, and results are not presented. The second one performs the real-time remote monitoring of diabetes patients. The smart sensors used are ECG, blood glucose, and movement sensors. The fog/edge device used is a smartphone. The AI method adopted is a decision tree for diabetes risk prediction classification. Only the cloud was used for centralized storage and fog devices to perform data collection, pre-processing, feature extraction, data compression, security, and analytics. The third one performs stress prediction and classification for monitoring heart rate and diabetes. The smart sensors use various wearable body sensors and embedded devices. The fog/edge device is a computer. The AI method adopted is a DL classifier for predicting early symptoms of type 2 diabetes.
- Ref. [35] proposes a framework model with three main layers, including the sensor layer, edge layer, and cloud layer. In the summary of AI-based edge-assisted smart healthcare solutions, none were found for diabetes application cases. This work does not include a study case.
- Ref. [37] discusses the current state-of-the-art smart healthcare systems. In the summary of works on smart health monitoring systems based on wearable devices, none were found for diabetes. The communication media/protocol only describes works using Wi-Fi, HTTP, MQTT, and Bluetooth hc-06. None were found to use 5G. In the summary of works on smart health monitoring systems based on smartphones, there exists only one for glucose levels through video analysis. In the summary of works on diabetes detection frameworks in IoT health environments, eight were found. In all works, the AI task was classification. In the works, no user prototype was shown, detailed data collection procedures were not mentioned, a glucose sensor and Arduino could not be operated at the same time, the latency was comparatively high, and real-time cases were not found.
- Ref. [50] introduces an edge-of-things computing framework for secure and smart healthcare surveillance services and a case study with electrocardiogram ECG data. The architecture comprises four main entities: community members (includes patients and wired/wireless sensors); a medical services gateway and an IoT gateway to collect data, perform local processing, encrypt health data, and perform transmission to the cloud; a cloud-enabled database to store encrypted healthcare data; and an abnormality detection model to analyze the encrypted data. Thus, the proposed system architecture shows an edge computing layer that is internally composed of two edge devices: the medical services gateway and the IoT gateway. The biosignal data are captured via sensors and transmitted over a 5G network to the medical services gateway. After that, this edge device again transmits the data over a 5G network to the IoT gateway, which finally sends the data to the cloud.

From the experiments with 50 virtual ENLs, it is observed that the proposed architecture presents a low throughput and a high average response time, which struggles to send and process high data volume, which results in congestion, an error-rate increase, and poor architecture performance. In contrast, experiments with 20 virtual ENLs observed that a high throughput and low latency produce a responsive and efficient architecture, and ENLs experience improved performance. Therefore, several factors contribute to low throughput and a high average response time: server overload, 5G network connectivity issues, or high network traffic. If data must travel a long distance between the client and server or if there are issues with the network infrastructure, they can result in delays. If the server becomes suddenly inundated with requests, it may struggle to process them all on time, leading to increased latency.

However, this study faced some limitations. First, the study case was only a preliminary work. Second, the metric performance of the best AI models presented in the study case requires improvement and extensive experiments with the size of the dataset samples increased and the diversification of the volunteers extended. Third, experimental results can be enhanced by introducing a more diverse participant sample, as the current dataset consists of only 12 volunteers. Fourth, the architecture has not been tested under varied environmental conditions and differing urban and rural network infrastructures, which would help generalize the results to broader contexts.

Regarding security, the work implements the OAuth2.0 protocol and follows the OAuth 2.0 Security Best Current Practice described in [51]. Regarding data privacy, the proposed work follows the Brazilian General Personal Data Protection Law (LGPD), Law No. 13709/2018, which protects the fundamental rights of freedom and privacy and the free formation of each individual’s personality.

Future research works envision edge-IoT with embedded AI (edge-AI-IoT) and federated learning between edge-AI-IoT devices to deliver a scalable and highly optimized s-health application. We can list a set of advantages for such a device at a large-scale application, such as the following:

- Increased speed and lower latency eliminate the need for back-and-forth communication with the cloud. An edge-AI-IoT saves data-processing and bandwidth costs by running on-device, reducing power consumption and prolonging battery life.
- Federated learning enables edge-AI-IoT to share end-user information without the need to take their data to central cloud storage and to access the appropriate set of data locally and experience learning activities without disruptions.
- AI models are on the device, and there is no need to send sensitive data to the cloud for processing. Personal data remain on personal devices.
- AI models are trained on user inputs and are thereby optimized for individual users. Furthermore, AI functions are available offline and can be accessed at any time.
- Federated learning allows edge-AI-IoT devices to learn a shared prediction model collaboratively. Since prediction takes place on the edge device, real-time prediction is feasible. Thus, the prediction method works even though there is no internet connection because the models are stored on the device.

Deploying embedded AI in a resource-constrained edge device requires addressing acceleration and compression. The current CPU/GPU acceleration algorithms are divided into adjusting the task scheduling strategy, enhancing CPU-GPU parallel computing efficiency, and strengthening GPU utilization. For model size compression, two possible methods for deployment and inference allow for speeding up and energy saving without significant accuracy losses. Quantization to reduce the number of bits can be used for floating-point numbers, maintaining model accuracy as much as possible, and pruning to reduce redundant data in the model by determining the importance of each unit and removing unimportant parts.

## Figures and Tables

**Table 1:** Baseline algorithms’ performance with reduced features trained and tested on a dataset of twelve volunteers. CB-R*: best model; ABR**: second model; RFR***: third model.

Model	Features	MSE		RMSE		$R^{2}$	
		Training	Test	Training	Test	Training	Test
MLR	35	547.54	2353.33	23.40	48.51	0.53	–1.06
SVR	20	555.28	1095.70	23.56	33.10	0.52	–0.08
KNN-R	15	971.31	1375.59	31.17	37.09	0.16	–0.20
DTR	15	728.72	39.60	26.99	39.59	0.37	–0.37
B-DTR	15	481.01	1282.91	21.93	35.81	0.59	–0.26
RFR***	20	212.95	960.34	14.59	30.98	0.82	0.16
ABR**	15	186.19	934.03	13.65	30.56	0.84	0.18
GBR	15	0.03	1336.85	0.17	36.56	0.99	–0.17
XGBR	20	0.11	1399.40	0.34	37.41	0.99	–0.22
MLP	15	942.75	1443.72	30.70	37.99	0.18	–0.26
CB-R*	**15**	**218.91**	**782.30**	**14.80**	**27.97**	**0.81**	**0.31**
CNN-1D	15	903.86	1057.93	30.06	32.53	0.22	0.09

**Table 2:** Comparison of non-invasive glucose prediction approaches of wearable sensors available in the literature with the best AI algorithm of the proposed work. NIR: near-infrared spectroscopy; FFNN: feed-forward neural network; RR: ridge regression; MATS: moving average time series; GD: gradient boosting; MAPE: mean absolute percentage error; MAE: mean absolute error; MARD: mean absolute relative difference.

Reference	Technology	AI Algorithm	Performace Metric
[45]	NIR	FFNN	MAPE (%)—1.94 MAE (mg/dL)—2.49 MSE (mg/dL)—9.16 RMSE—3.02 $R^{2}$—0.99
[46]	NIR	RR	Fingertip Wrist MAE—0.15, 0.66 MSE—0.2287, 0.006 $R^{2}$—0.9902, 0.9996
[47]	NIR	FFNN	MARD—12.50% $R^{2}$—0.97
[48]	Impedance spectroscopy	MATS	RMSE—14.61 MAPE—0.11
[49]	Bioimpedance	GB	MARD—17.9%
**Our** **work**	**Bioimpedance**	**CB-R**	**MSE—218.91/782.30** **RMSE—14.80/27.87** **$R^{2}$—0.81/0.31**

## Data Availability

Data are available upon request.
